# Study protocol: Couples Partnering for Lipid Enhancing Strategies (CouPLES) – a randomized, controlled trial

**DOI:** 10.1186/1745-6215-10-10

**Published:** 2009-02-06

**Authors:** Corrine I Voils, William S Yancy, Stacey Kovac, Cynthia J Coffman, Morris Weinberger, Eugene Z Oddone, Amy Jeffreys, Santanu Datta, Hayden B Bosworth

**Affiliations:** 1 Center for Health Services Research in Primary Care, Durham Veterans Affairs Medical Center, Durham, North Carolina, USA; 2 Department of Medicine, Duke University Medical Center, Durham, North Carolina, USA; 3 Department of Biostatistics and Bioinformatics, Duke University Medical Center, Durham, North Carolina, USA; 4 Department of Health Policy and Administration, University of North Carolina at Chapel Hill, Chapel Hill, North Carolina, USA

## Abstract

**Background:**

Almost 50% of Americans have elevated low-density lipoprotein cholesterol (LDL-C). The behaviors required to lower LDL-C levels may be difficult to adhere to if they are inconsistent with spouses' health practices, and, alternatively, may be enhanced by enlisting support from the spouse. This trial extends previous trials by requiring spouse enrollment, teaching spouses how to provide emotional and instrumental support, allowing patients to decide which component of the intervention they would like to receive, and having patients determine their own goals and action plans.

**Methods:**

Veteran outpatients with above-goal LDL-C (N = 250) and their spouses are randomized, as a couple, to receive printed education materials only or the materials plus an 11-month, nurse-delivered, telephone-based intervention. The intervention contains four modules: medication adherence, diet, exercise, and patient-physician communication. Patients decide which modules they complete and in which order; modules may be repeated or omitted. Telephone calls are to patients and spouses separately and occur monthly. During each patient telephone call, patients' progress is reviewed, and patients create goals and action plans for the upcoming month. During spouse telephone calls, which occur within one week of patient calls, spouses are informed of patients' goals and action plans and devise strategies to increase emotional and instrumental support.

The primary outcome is patients' LDL-C, measured at baseline, 6 months, and 11 months. Linear mixed models will be used to test the primary hypothesis that an 11-month, telephone-based patient-spouse intervention will result in a greater reduction in LDL-C as compared to printed education materials. Various process measures, including social support, self-efficacy, medication adherence, dietary behavior, and exercise, are also assessed to explain any change, or lack thereof, in LDL-C.

**Discussion:**

Given the social context in which self-management occurs, interventions that teach spouses to provide instrumental and emotional support may help patients initiate and adhere to behaviors that lower their LDL-C levels. Moreover, allowing patients to retain autonomy by deciding which behaviors they would like to change and how may improve adherence and clinical outcomes.

**Trial Registration:**

The ClinicalTrials.gov registration number is NCT00321789.

## 

Coronary heart disease (CHD) is the leading cause of death in the United States, resulting in more than 500,000 deaths and an additional 500,000 nonfatal heart attacks annually.[1,2] One major modifiable risk factor for CHD is elevated low-density lipoprotein cholesterol (LDL-C). Every 1% reduction in LDL-C is accompanied by a 1% reduction in individuals' short-term risk of a major coronary event.[2] Consequently, current guidelines designate LDL-C as the primary target of lipid-lowering therapy.[2] Despite the proven efficacy of lifestyle modifications and lipid-lowering medications, almost half of American adults have borderline high or high LDL-C, defined as 130 mg/dL or above.[2] Therefore, novel interventions are needed to lower LDL-C levels.

Patient interventions have proven effective for increasing adherence to behaviors that would lower LDL-C levels.[3,4] However, changes are typically short lived, with less healthy behaviors returning after brief periods of time. One reason for nonadherence is that recommended lifestyle behaviors may be inconsistent with those of one's social network members.[4,5] Moreover, patients may have difficulty adhering to dietary changes when they are not involved in cooking meals or grocery shopping.[6] What is needed, then, are interventions that can enhance the social support provided by spouses/significant others in order to help patients adhere better to treatment recommendations.

Several trials have shown that spouses can help patients lower their cholesterol.[7,8] However, these studies have some limitations. First, they have targeted a young population.[9] or women. [10,11] Thus, these studies do not generalize to the VA setting, which consists of primarily older males for whom the female spouse is the primary meal planner. Second, these studies have evaluated relatively short-term interventions (i.e., less than 6 months).[9,11] Third, previous studies have simply encouraged emotional support, rather than *teaching *spouses how to provide better emotional *and instrumental *support. [10,12] This is important because spouses may do things to decrease treatment adherence (e.g., nagging). Fourth, previous studies have not allowed patients to create their own goals and action plans (i.e., steps for achieving those goals). This is important because people who are not ready to make a behavior change – especially one imposed on them – are unlikely to be adherent.[13] Finally, these studies have targeted only one component of the treatment regimen at a time – either lifestyle changes or medication adherence.[9,12]

Our proposed intervention, the CouPLES trial, extends interventions tested in previous studies in several ways. We will: (1) target elderly male veterans, a group at increased risk for dyslipidemia and associated morbidity; (2) assess both short-term (6 months) and long-term (11 months) effects of the intervention; (3) employ a multi-component intervention that includes modules for medication, diet, exercise, and patient-physician communication – all important components of treatment for dyslipidemia; (4) have patients create goals and action plans for whichever behavior they would like to change; and (5) teach spouses strategies to provide support that can enhance patients' adherence to healthy behaviors. In this paper, we describe the study design, recruitment strategy, and analytical techniques of a clinical trial to test this state-of-the-art CouPLES intervention.

## Methods

### Setting and overall study design

This randomized controlled trial is being conducted at the Durham Veterans Affairs Medical Center (VAMC) and was approved by the Durham VAMC Institutional Review Board. Written informed consent was obtained from the patient for publication of this case report and accompanying images. A copy of the written consent is available for review by the Editor-in-Chief of this journal. Patients are recruited from the primary care clinics, which are staffed by Internal Medicine faculty physicians, house staff physicians, physician assistants, and nurse practitioners. Eligible patients and their spouses are randomized, as a couple, to receive printed educational materials only (control arm) or printed educational materials plus the CouPLES intervention (intervention arm). During the study, all patients continue to receive usual care from their physician. The intervention is delivered over 11 months via a series of monthly telephone calls to both members of the couple. Patient outcomes are assessed at 6 and 11 months.

### Patient population and recruitment

Our study population consists of married veterans with above-goal LDL-C. According to current guidelines, a patient's LDL-C goal depends on the number of major risk factors and the estimated 10-year risk for CHD according to the Framingham risk score.[2] The three risk categories and corresponding LDL-C goals are shown in Table 1.

**Table 1:** Risk categories and LDL-C (mg/dL) goal

Risk Category	LDL-C Goal
High: CHD and risk equivalents*	<100
Medium: No CHD, ≥ 2 risk factors**	<130
Low: No CHD, 0–1 risk factor	<160

LDL-C: low-density lipoprotein cholesterol. CHD: coronary heart disease.

*Risk equivalents: diabetes, clinical forms of atherosclerotic disease, and multiple risk factors that confer a 10-year risk for CHD > 20% according to the Framingham risk score. **Risk factors: hypertension (blood pressure ≥ 140/90 mmHg or on antihypertensive medication), cigarette smoking, high-density lipoprotein cholesterol < 40 mg/dL, age (men ≥ 45 years, women ≥ 55 years), and family history of premature coronary heart disease (present in male first degree relative < 55 years or female first degree relative < 65 years).

**Figure 1:**
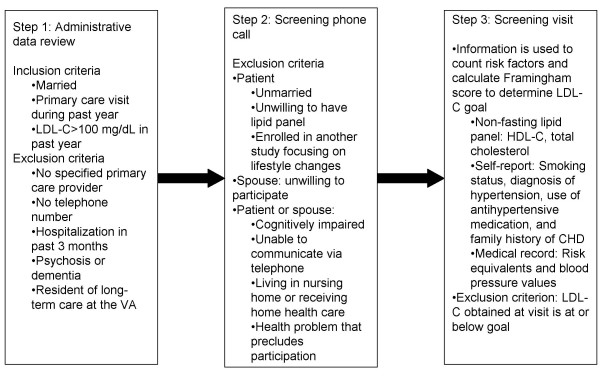
**Steps in identifying potentially eligible patients**.

We identify potential participants via a three-step process (Figure 1). In Step 1, we use VA administrative databases to identify married patients who have had a primary care visit and a lipid profile with LDL-C >100 mg/dL in the previous 12 months. We chose a cutoff of 100 mg/dL because that is the lowest possible guideline-recommended LDL-C goal. We also use the databases to exclude patients who meet any of the exclusion criteria listed in Step 1 of Figure 1.

In Step 2, patients who have an upcoming clinic appointment receive a recruitment letter, followed by a screening telephone call. Based on responses given during the screening telephone call, patients who meet any of the exclusion criteria listed Step 2 of Figure 1 are excluded. Patients passing Step 2 are scheduled for an in-person screening visit with a research assistant (RA). Spouses are also required to attend; in most cases, spouses have been able to come at the same time as patients. This may not be true in a younger population that is more likely to be employed and caring for young children.

In Step 3, self-reported data and laboratory results obtained during the screening visit, combined with medical record data, are used to count patients' risk factors and calculate their Framingham risk scores (see Figure 1, Step 3). This information is used to determine patients' risk categories, as defined in Table 1, and their individual LDL-C goals based on the risk categories. Patients with above-goal LDL-C are eligible for enrollment. Eligible couples are randomized, as a pair, to the intervention or control arm. The randomization is stratified on patient race (White and non-White) and risk category (collapsed from three categories to two – high and medium/low – due to the low prevalence of the low-risk category). This will allow us to test for differential effects of the intervention on different race and risk groups, which will inform future interventions.

The screening visit also serves as the baseline visit for eligible patients. At this visit, patients and spouses provide written informed consent, complete baseline measures, and receive printed educational materials. After the visit, the research nurse who delivers the CouPLES intervention places a telephone call to inform patients of their laboratory results, eligibility, and when eligible, randomization assignment. During this telephone call as well as the intervention telephone calls preceding follow-up assessments, the nurse instructs couples not to reveal their study assignment to the RA so that the RA will be blinded for outcome assessments. Each couple receives $40 for each of the three visits.

### CouPLES intervention

#### Overview

**Figure 2:**
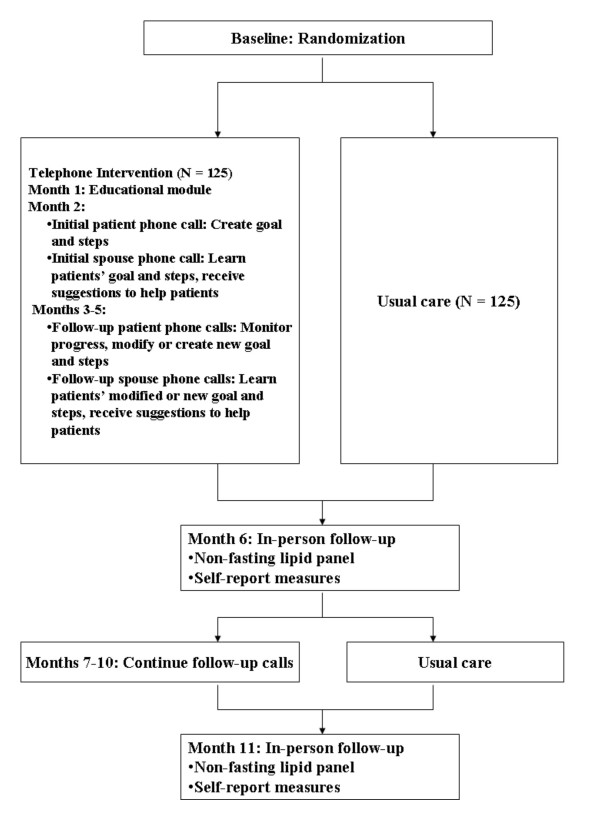
**Intervention flowchart**.

The CouPLES intervention takes place over 11 months (Figure 2). A research nurse delivers the CouPLES intervention because clinical knowledge and judgment are required (e.g., for issues with medications or comorbidities). Prior to enrollment, the nurse received didactic training from a clinical psychologist (S.K.) on the basic principles of motivational interviewing, including asking open-ended questions, learning how to use reflective listening, and learning to identify and elicit change talk from a patient. The nurse also learned how and when to reinforce patients and to express optimism about their ability to change. The nurse received feedback on several practice intervention calls to both patients and spouses from C.V. and S.K. During the trial, 5% of calls are recorded and reviewed to assure intervention fidelity, and feedback is provided as necessary.

The research nurse conducts all intervention telephone calls with patients and spouses separately. During the month 1 telephone call, patients and spouses receive an educational module. During the month 2 telephone call, patients choose one of four topical modules and create corresponding goals and action plans. During subsequent monthly telephone calls, which occur every month except months 6 and 11 when outcomes are assessed, patients' progress is reviewed, and patients revise or create new goals and action plans for the same or a different module. During all spouse telephone calls, spouses are informed of patients' goals and action plans and are provided with strategies to provide better emotional and instrumental support to help increase patient adherence. For example, spouses may exercise with patients or prepare their lunch so that they do not have to eat at a restaurant.

#### Module content

At month 1, the nurse delivers an educational module that reviews the importance of blood cholesterol to health, treatments for dyslipidemia, and principles of self-management. In the spouse educational call, the nurse also reviews behavioral principles for providing instrumental and emotional social support. Although this information is provided in the printed materials distributed at baseline, the nurse reviews it to ensure that all intervention patients will receive the information, regardless of levels of health literacy, and so that the nurse can answer any questions or clarify the information. Below, we describe the content of each of the four topical modules and processes for delivery.

#### Diet

The educational materials include a handout on portion sizes and the National Heart, Lung, and Blood Institute's cookbook *Keep the Beat: Heart-Healthy Recipes*. We designed the diet module to be applicable regardless of whether the patient or spouse is the primary meal preparer in the home, and we provide different suggestions for people who do and do not cook. The nurse asks spouses if they plan to make the same dietary changes as the patients and provides appropriate suggestions.

#### Exercise

Patients receive a handout emphasizing the importance of exercise to health and suggesting ways to start an exercise program, stay motivated, deal with pain, and prevent overexertion. As in the diet module, the nurse asks spouses if they plan to exercise with patients and provides appropriate suggestions.

#### Medication adherence

This module will only be applicable to patients who are on lipid-lowering medications (which is not an inclusion criterion) and who need assistance to increase medication adherence. We provide information sheets on lipid-lowering medications that include dosages, dosing instructions, the most common side effects, and important warnings, all written at an eighth grade reading level. Suggestions to spouses correspond to patients' goals and may include ordering refills, encouraging patients to contact their pharmacist or physician about side effects, and helping patients prepare pill boxes.

#### Patient-physician communication

The written materials suggest ways patients can improve the quantity and quality of communication with their providers. The nurse provides practical suggestions for bringing concerns to the doctor's attention and encourages role-playing with spouses to increase patients' self-efficacy. The nurse also helps patients problem-solve and set goals that are reasonable to accomplish at their next clinic visit using methods such as role-playing.

#### Module delivery

During the initial patient telephone call, patients choose one of the four topics. The nurse asks open-ended questions about what they have tried in the past, what has worked, and what has not worked. The nurse also uses reflective listening to help patients establish possible goals related to the topic chosen. The nurse asks patients to rank each possible goal in order of importance to determine which goals will be pursued in the upcoming month. Next, the nurse uses open-ended questions to elicit from patients how they will attempt to make the behavior changes necessary to reach that month's goals and how to integrate the spouse in the plan; these specific behaviors are designated as action plans. For each action plan, patients are asked to rate their self-efficacy on a 1–10 scale (1 = *not at all confident*, 10 = *very confident*). Patients reporting < 7 are asked to revise their action plans and/or goals until ratings ≥ 7 are provided, as patients will be more likely to accomplish their goals.[14] Patients are asked to record that month's goals and action plans on provided goal sheets, which they can refer to anytime.

The follow-up patient calls take place 3–5 weeks after the initial calls (i.e., there is a 2-week window during which calls must occur, otherwise the call is skipped). During these calls, patients are asked whether they met their goals. Patients who met their goals are reinforced and encouraged to create additional goals and action plans. Patients who did not meet their goals are encouraged to modify their original goals and action plans or create new one(s).

All spouse calls occur within one week of the patient calls. During these calls, spouses are informed of whether patients indicated that they met their goals and about patients' new or revised goals and action plans. Spouses are encouraged to think of ways to help patients achieve their new or revised goals and, when necessary, are provided with suggestions.

This intervention takes into account patients' motivation to change specific behaviors by allowing them to determine which modules they receive and the order in which they receive them. This way, patients address the areas about which they are most motivated to make behavioral changes. Some patients may not wish to complete one or more modules. Because this is a patient-centered intervention, the protocol is flexible so that modules can be repeated or omitted.

#### Information technology for intervention delivery

Two customized database applications were created: one for the delivery of the CouPLES intervention, and one for the tracking of all related data. Once couples are randomized to the intervention, relevant information is automatically transferred from the study tracking database to the intervention application database to facilitate intervention delivery. This information includes participants' names, contact information, demographic data, and contact information for patients' VA primary care provider.

Once couples' information enters the intervention application database, the nurse can track the date on which the next telephone intervention call is due to both patients and spouses, as well as dates of previous intervention telephone calls. Several other important features of the application facilitate tracking of telephone calls. First, a calendar notes the patient-specified best days and times to be reached. This information is obtained by the nurse during the educational telephone call and allows calls to be made when the participant is most likely to be available. The application provides lists of all patients and spouses due for telephone calls and notes those who are currently available. Second, the application allows the nurse to document the number of missed calls, time periods when the participant will be on vacation or otherwise unavailable, and any other notes regarding availability. Third, the application automatically records the duration of each telephone call. This information will be used in the economic evaluation and will provide information regarding the time required to implement this type of intervention in a real-world clinical setting.

**Figure 3:**
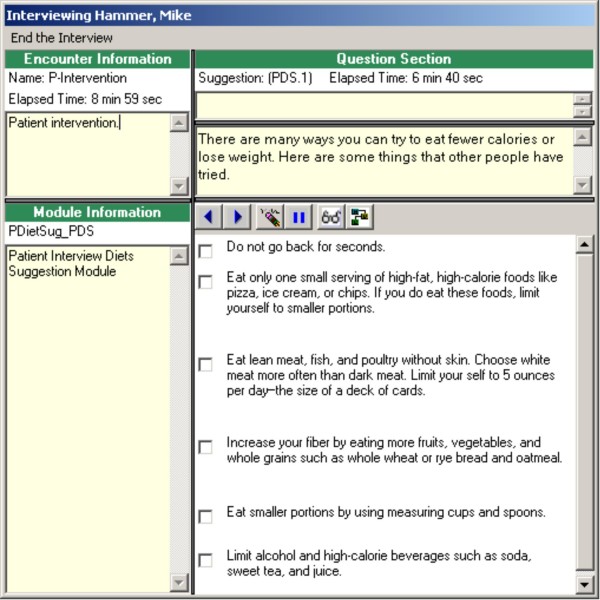
**Patient telephone script from diet application**.

**Figure 4:**
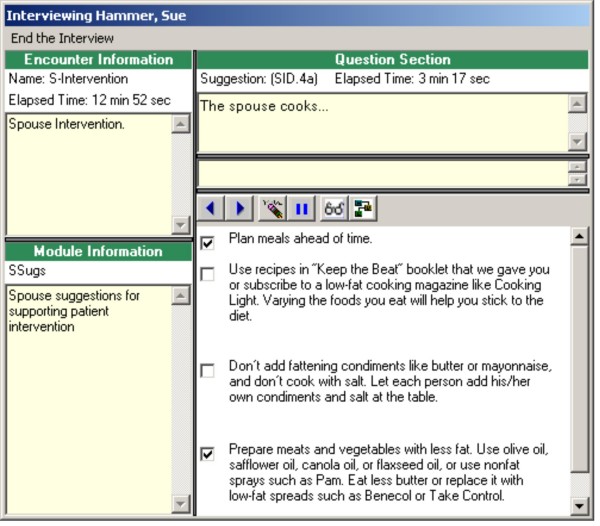
**Spouse telephone script from diet application**.

**Figure 5:**
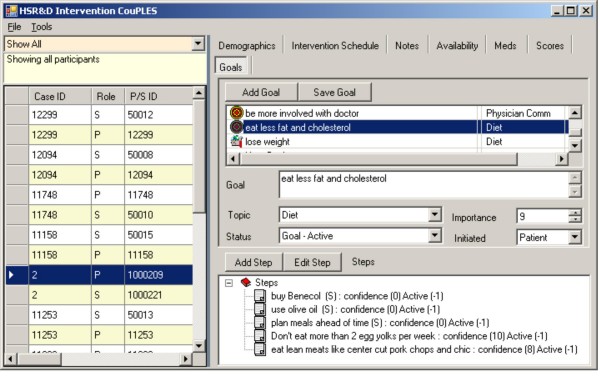
**Patient goals from intervention delivery application**.

In addition to tracking telephone call schedules and data, the application facilitates implementation of the intervention. For each topical module, the application provides scripts for the nurse to follow during each telephone call. These scripts contain summary points, questions for patients and spouses, and suggested behaviors that patients and spouses could designate as action plans to follow over the next month (Figures 3 and 4 display dietary suggestions from the patient and spouse diet modules; names are fictitious). Most importantly, the application provides an interface for documenting and tracking patients' goals and action plans (Figure 5 displays goals and action plans for a fictitious patient). For a selected topic, all goals and action plans are displayed, and the software shows which goals are active, inactive, or complete at the time of the telephone call. The application also tracks patients' self-efficacy at the time action plans are initiated, and whether the goal was initiated by the participant or initially suggested by the nurse.

#### Study measures

Control variables are assessed at baseline. The primary, secondary, and process measures are assessed at baseline, 6 months, and 11 months.

#### Control variables

Demographic variables (age, sex, race, education, financial stress, employment status) and clinical variables (cholesterol medication use, frequency of cholesterol testing, alcohol and tobacco use, antihypertensive medication use, family history of CHD) are assessed at via self-report. Health literacy is assessed with the Short Test of Functional Health Literacy in Adults (S-TOFHLA).[15] Comorbidities are assessed with a self-report checklist.[16]

#### Primary outcome

LDL-C is measured directly using a non-fasting lipid profile. We chose a non-fasting test because it reduces patient burden and therefore may increase our enrollment rate. The blood sample is analyzed with an LXi from Beckman Coulter. The coefficient of variation for LDL-C ranges from 3.3 % at 106 mg/dL to 4.1 % at 49 mg/dL.

#### Secondary outcomes

Dietary behavior is assessed with the Block Brief 2000 Food Frequency Questionnaire (FFQ).[17] Exercise is assessed with the Community for Healthy Activities Model Program for Seniors (CHAMPS) questionnaire.[18] Medication adherence is assessed with the Med-Out index, which is calculated for each lipid-lowering medication.[19]

#### Process measures

Several measures are collected to determine what is responsible for LDL-C change. These include cholesterol knowledge; self-efficacy and outcome expectancies for each of the behaviors; spouse support for dietary change.[20] and exercise;[21] satisfaction with the dyadic relationship;[22] amount of patient-physician communication;[18] and intervention intensity (i.e., how many phone calls were completed).

### Sample size and statistical power

The primary hypothesis is:

(H1) An 11-month, telephone-based patient-spouse intervention will result in a greater reduction in LDL-C as compared to printed education materials.

In our sample size calculations, we assume no change for patients who receive only printed education materials and a 7% reduction in LDL-C (based on a 2-month pilot study using the same intervention materials) for intervention patients from baseline to 11 months. Based on a standard deviation adjusted to account for correlation between repeated measurements of the outcome, and using an independent samples t-test with a 0.05 two-sided significance level and 80% power, a sample size of 100 patients per group is required to detect a 9.4 point (7%) difference in change of LDL-C values between the intervention and control groups. Because we assume an approximate dropout rate of 20%, we will recruit approximately 125 couples per arm.

### Primary analyses

The unit of analysis is the patient. Because LDL-C, a continuous variable, is assessed at baseline, 6 months, and 11 months, we will use a linear mixed-effects model (LMM).[23] Fixed effects in the model will include intervention group, time, and the interaction between these two variables. This will enable us to determine the effects of the intervention at both 6 and 11 months. Patient-level random effects will be included in the model to account for correlations between patients' repeated measures over time. Primary analyses will be conducted as intent-to-treat, and sensitivity analyses will examine the implications of the intent-to-treat assumption.

### Secondary analyses

The secondary hypotheses are that patients who receive the intervention will have significantly greater medication adherence and exercise, and consume fewer calories, compared to patients who only receive printed education materials. Because these outcomes are continuous and are assessed at three time points for each patient, we will use LMMs. We are also interested in changes in self-efficacy, spousal support, and patient-physician communication. We will examine whether changes in these process measures are responsible for changes in LDL-C at both 6 and 11 months using currently accepted methods.[24,25]

### Economic evaluation

The period of analysis will be the 11-month intervention period. The base-case cost analysis will be conducted from the VA's perspective. We will estimate the average annual cost of the intervention per patient as well as the cost of resource utilization, focusing on primary care visits and cholesterol medications. Additionally, if the intervention is more effective than printed education materials, we will assess the incremental cost-effectiveness of the intervention. For the cost-effectiveness analysis, we will use percent reduction in LDL-C as the effectiveness measure.

## Discussion

Recommended therapies for LDL-C reduction are only effective if patients are adherent.[26,27] Unfortunately, patient adherence to CHD prevention treatment is sub-optimal, resulting in LDL-C levels above guideline-recommended goals. Increasing the support provided to patients is likely to improve their adherence to treatment recommendations. In the CouPLES trial, we are assessing the effectiveness of teaching patients and spouses strategies to help increase patient adherence, which in turn should lower LDL-C levels.

The CouPLES intervention is novel in a number of respects. First, to our knowledge, this is the first patient-spouse intervention that addresses all major components of the lipid-lowering regimen. Second, rather than receiving topical modules in a prescribed order, patients can decide which modules they would like to receive and how often. They can choose one topic for the entire duration of the study or choose any combination of topics. Third, we use multiple modes to deliver the information, including printed educational materials, telephone calls, and reinforcement by spouses. This is important because patients typically receive information in one format (usually verbally) in a single clinical encounter and are likely to misunderstand or forget it. Fourth, the nurse teaches spouses how to provide emotional and instrumental support rather than just suggesting that they should be involved. Fifth, the nurse tailors suggestions according to whether spouses intend or do not intend to make the same lifestyle changes as patients. Finally, the intervention is delivered by telephone, which has several advantages over face-to-face strategies, including lower cost and wider reach.[28,29] Telephone interventions allow a nurse to follow a much larger panel than would be possible with in-person interventions. For this reason, we will assess the cost effectiveness of the intervention to help determine the resources that would be necessary to implement it.

## Conclusion

CHD is associated with significant healthcare costs, with nearly $130 billion in direct and indirect costs in 2003.[1] The expected increase in prevalence of CHD over the next several decades will result in an increased burden for many health care systems. Despite the known risk of elevated LDL-C, patients are not adhering to the behaviors necessary to achieve optimal LDL-C levels. Given that the latest recommendations have even lower goals for LDL-C,[30] more effective interventions are needed. If the CouPLES intervention proves effective and low-cost, it could help reduce future healthcare costs associated with the long-term effects of elevated LDL-C levels.

## Abbreviations

CHAMPS: Community for Healthy Activities Model Program for Seniors; CHD: coronary heart disease; CouPLES: couples partnering for lipid enhancing strategies; FFQ: food frequency questionnaire; LDL-C: low-density lipoprotein cholesterol; LMM: linear mixed model; mg/dL: milligrams per deciliter; RA: research assistant; S-TOHFLA: Short Test of Functional Health Literacy in Adults; VA: Veterans Affairs; VAMC: Veterans Affairs Medical Center.

## Competing interests

The authors declare that they have no competing interests.

## Authors' contributions

All authors participated in the conception and design of the trial. CV drafted the protocol, created the written intervention materials and telephone scripts, trained the staff to conduct the study, and wrote the manuscript. WY provided content expertise for the intervention materials and telephone scripts and oversees adverse event reporting and clinical issues. SK provided content expertise for the intervention materials and trained the nurse to conduct the intervention. CC conducted the power analysis and will conduct and supervise statistical analyses. AJ developed the algorithm for calculating Framingham scores and pulling data into the tracking database and will conduct statistical analyses. SD will conduct all economic analyses. MW, EO, and HB provided input on the telephone scripts. HB, the Co-Principal Investigator, provides oversight on all aspects of the study.
